# Substitution of dietary fish oil with plant oils is associated with shortened mid intestinal folds in Atlantic salmon (*Salmo salar*)

**DOI:** 10.1186/1746-6148-10-60

**Published:** 2014-03-07

**Authors:** Torfinn Moldal, Guro Løkka, Jannicke Wiik-Nielsen, Lars Austbø, Bente E Torstensen, Grethe Rosenlund, Ole Bendik Dale, Magne Kaldhusdal, Erling Olaf Koppang

**Affiliations:** 1 Norwegian Veterinary Institute, Post Box 750 Sentrum, 0106 Oslo, Norway; 2 Norwegian School of Veterinary Science, Post Box 8146 Dep, 0033 Oslo, Norway; 3 National Institute of Nutrition and Seafood Research, Post Box 2029 Nordnes, 5817 Bergen, Norway; 4 Skretting ARC, Post Box 48, 4001 Stavanger, Norway

**Keywords:** Atlantic salmon, Fatty acids, Fish oil, Inflammation, Intestine, Morphometric analyses, Plant oils, Real-time PCR

## Abstract

**Background:**

Fish meal and fish oil are increasingly replaced by ingredients from terrestrial sources in the feeds for farmed salmonids due to expanding production and reduced availability of marine feed raw material. Fish oil that is rich in n-3 polyunsaturated fatty acids is considered beneficial to human health in general and to prevent intestinal inflammation and carcinogenesis in particular. In contrast, n-6 fatty acids that are present in many vegetable oils have been associated with increased risk of colitis and colon cancer in rodents and humans, as well as lowered transcription levels of certain stress and antioxidant-related genes in Atlantic salmon.

The aim of the present study was to investigate the intestinal health in Atlantic salmon fed with different vegetable oils as partial substitutes of fish oil in the diet. A feed trial lasting for 28 weeks included one reference diet containing fish oil as the sole lipid source and three diets where 80% of the fish oil was replaced by a plant oil blend with either olive oil, rapeseed oil or soybean oil as the main lipid source. These plant oils have intermediate or low n-3/n-6-ratios compared to fish oil having a high n-3/n-6-ratio. The protein and carbohydrate fractions were identical in all the feeds.

**Results:**

Morphometric measurements showed significantly shorter folds in the mid intestine in all groups fed vegetable oils compared to the group fed fish oil. In the distal intestine, the complex folds were significantly shorter in the fish fed soybean oil compared to the fish fed rapeseed oil. Histological and immunohistochemical examination did not show clear difference in the degree of inflammation or proliferation of epithelial cells related to dietary groups, which was further confirmed by real-time RT-PCR which revealed only moderate alterations in the mRNA transcript levels of selected immune-related genes.

**Conclusions:**

Shortened intestinal folds might be associated with reduced intestinal surface and impaired nutrient absorption and growth, but our results suggest that partial substitution of dietary fish oil with vegetable oils does not have any major negative impact on the intestinal health of Atlantic salmon.

## Background

Salmonids are indigenous carnivores, and fish meal and fish oil have traditionally been the main ingredients in feed for farmed Atlantic salmon (*Salmo salar*) and rainbow trout (*Oncorhynchus mykiss*). Due to limited sources of marine raw materials, a fast growing aquaculture industry and increased focus on sustainability, the salmon farming industry has searched for alternative feeds [1-3]. The inclusion of plant-derived materials in fish feed may be beneficial from an economic and ecological point of view, but used without carefully considering the fish minimum requirements or upper tolerable levels of certain nutrients or anti-nutrients, it can unfortunately also cause adverse effects with regard to fish health, nutritive value and the consumers’ acceptance [1].

In the early years of salmonid farming, proteins constituted more than half of the feed content, while the lipid fraction was as low as 10%. These figures have changed during the last decades as the protein level has decreased to less than 40%, while the lipid fraction has increased till about 35% [4]. Fish oil production requires 2-5 times more industrial fish than production of the same weight of fish meal [5], and replacement of fish oil with vegetable oils is hence of growing interest both from an economic and sustainability viewpoint.

**Figure 1:**
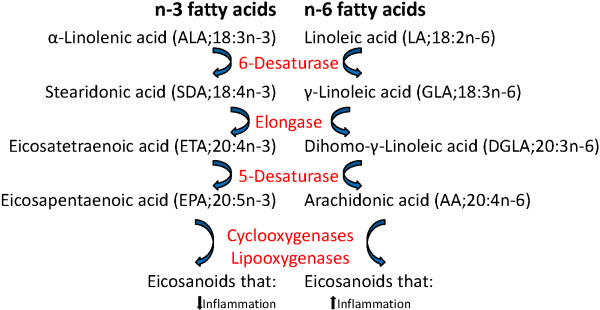
**The metabolism of n-3 and n-6 fatty acids and eicosanoids.** The same enzymes are involved in the metabolism of n-3 and n-6 fatty acids and synthesis of eicosanoids, but the biological properties of the eicosanoids are different.

Vegetable oils may contain high levels of n-6 polyunsaturated fatty acids (PUFAs) such as linoleic acid (LA, 18:2n-6) that can be further metabolized to arachidonic acid (AA; 20:4n-6). In contrast, fish oils are rich in n-3 PUFAs such as docosahexaenoic acid (DHA, 22:6n-3) and eicosapentaenoic acid (EPA, 20:5n-3) (Figure 1). In mammals, dietary n-3 fatty acids supplant the AA in inflammatory cell membranes [6] and therefore decrease the availability of the major precursor of pro-inflammatory eicosanoids as the same enzymes are involved in the metabolism of n-3 and n-6 fatty acids and further in the synthesis of eicosanoids [7]. Also in Atlantic salmon, dietary lipid has been shown to alter leucocyte phospholipid fatty acid composition and eicosanoid production [8], and increased n-6 levels in the feed gave increased n-6 fatty acids in leucocytes in an *ex vivo* study [9].

In mammals, a diet rich in n-6 fatty acids has been associated with increased risk of ulcerative colitis [10] and promotion of intestinal carcinogenesis [6,11], while a high intake of n-3 PUFAs is considered to be beneficial for health. Consumption of n-3 fatty acids has been shown to attenuate the dysbiosis and colitis caused by n-6 polyunsaturated fatty acid in mice [12] and to prevent and modulate a wide range of pathological conditions as cardiovascular diseases, diabetes and several inflammatory and neoplastic processes, including inflammatory bowel disease and colon cancer [13]. The n-3 fatty acids also inhibit the prostaglandin synthesizing enzyme cyclooxygenase-2 (COX-2) which is up-regulated during inflammation, the expression of the pro-inflammatory cytokines tumour necrosis factor-α (TNF-α) and interleukin-1 (IL-1) and the proliferation of lymphocytes as shown both *in vitro* and in rodent models [13-15].

Several studies have addressed the effects of vegetable oils as lipid sources in the feed on Atlantic salmon intestinal absorption [16], post-absorptive fates [17], feed uptake, growth rate, metabolism and nutrient content of the fish filet [2,18-22]. Whereas many studies have addressed the intestinal health of the fish when fish meal is replaced by different plant-derived proteins, and both soybean meal and pea protein concentrate have been shown to induce enteritis [23,24], there is a knowledge gap regarding the impact on intestinal health when fish oil is replaced by plant oils.

Complete substitution of fish oil with a plant oil blend containing rapeseed oil, palm oil and linseed oil in the feed induced lower transcription levels of certain stress and antioxidant-related genes in the intestine [25]. Another feed trial with the same oil blend partly substituting fish oil in combination with plant proteins at different inclusion levels demonstrated that in response to acute physiological stress, high levels of plant-derived dietary ingredients can enhance COX-2 induction and synthesis of pro-inflammatory eicosanoids in the intestine of salmon [26]. It has also been speculated whether inclusion of plant oils in the feed contributed to intestinal carcinogenesis in brood stock Atlantic salmon [27].

The aim of the present study was to investigate the morphology of the intestinal wall, the presence of antigen presenting cells and T lymphocytes, the proliferation pattern of epithelial cells, and the transcript levels of selected immune-related genes including relevant cytokines, major histocompatibility complex class II (MHC class II), cluster of differentiation 3ζ (CD3ζ), immunoglobulins, the intracellular receptor nucleotide-binding oligomerization domain-containing protein 2 (NOD2) and COX-2a in the intestine of Atlantic salmon when dietary fish oil was partially replaced by different vegetable oil blends with varying n-3/n-6-ratio.

## Methods

### Animal ethics, fish and feed

**Figure 2:**
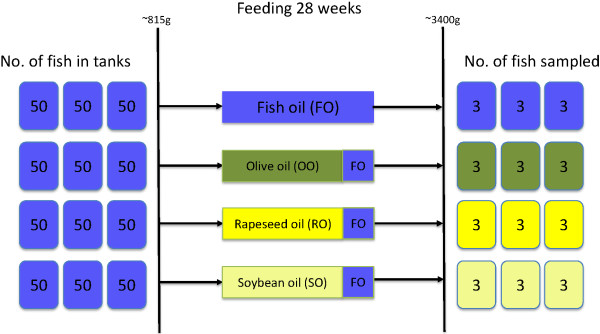
**The design of the feed trial.** Six hundred Atlantic salmon with mean initial weight of 815 ± 28 g were equally distributed into 12 tanks, and triplicate groups of fish were fed a diet with either fish oil as the sole lipid source or a diet where 80% of the fish oil was replaced by one of three vegetable oil blends with olive oil (OO), rapeseed oil (RO) or soybean oil (SO) as the main lipid source. The protein fraction was identical in all diets, and 70% of the proteins were plant-derived. The trial lasted for 28 weeks, and the mean final weight of all dietary groups was 3399 ± 76 g. Three fish in each tank were sampled by the end of the trial.

The feed trial was carried out at Skretting ARC Fish Trials Station that is approved by the Norwegian Animal Research Authority and was conducted according to current animal welfare regulations: FOR-1996-01-15-23 (Norway). Six hundred Atlantic salmon (*Salmo salar*) with mean initial weight of 815 ± 28 g were equally distributed into 12 tanks, and triplicate groups of fish were fed a diet with either fish oil as the sole lipid source or a diet where 80% of the fish oil was replaced by one of three vegetable oil blends with olive oil, rapeseed oil or soybean oil as the main lipid source (Figure 2). The n-3/n-6 ratio in the feed with fish oil as the sole lipid source was 5.3, while the n-3/n-6 ratios were 0.7, 0.9 and 0.3 in the diets where fish oil was largely substituted with olive oil, rapeseed oil and soybean oil respectively. The composition of the protein fraction was identical in all diets with 30% fish meal and 70% plant protein. The proximate composition of all diets was similar with 332-341 g kg^-1^ fat and 406-413 g kg^-1^ protein.

The fish were vaccinated intraperitonally (ALPHA JECT micro 6; PHARMAQ, Overhalla, Norway) to give protection towards furunculosis, vibriosis, cold-water vibriosis, winter ulcer disease and infectious pancreas necrosis one month prior to seawater transfer. The trial lasted for 28 weeks, and the mean final weight was 3399 ± 76 g with no significant difference between dietary groups. The fish were anesthetized with MS222 at a concentration of 7 g L^-1^ and euthanized according to regulations (Forskrift om drift av akvakulturanlegg §34. Avlivning av fisk). The design of the feed trial and feed composition is described in detail elsewhere [22].

### Histology and immunohistochemistry

**Figure 3:**
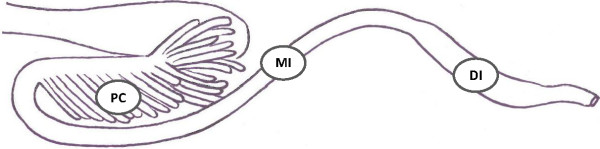
**Schematic drawing of the intestinal tract of Atlantic salmon.** The sampling sites are indicated as follows: PC = pyloric caeca, MI = mid intestine and DI = distal intestine.

For histological evaluation, tissues from the mid and distal intestine (Figure 3), also called first and second segment of mid intestine, respectively [28], from 24 fish in each dietary group (eight fish per tank) were fixed in 10% phosphate-buffered formalin for 24-48 hours. The tissues were routinely processed, embedded in paraffin and cut in 3 μm thick cross sections for the mid intestine and longitudinal sections (i.e. perpendicular to the macroscopically visible circular folds) for the distal intestine. Sections were routinely deparaffinized in xylene and rehydrated in graded alcohol baths before staining with hematoxylin and eosin (HE).

Formalin-fixed and paraffin-embedded tissues from the mid intestine and the distal intestine from nine fish in each dietary groups (three fish in each tank) were prepared for detection of antigen presenting cells expressing MHC class II and T lymphocytes expressing CD3ϵ by immunohistochemistry with salmon specific polyclonal rabbit antisera as previously described by Koppang *et al*. [29,30] with some modifications. Proliferating cells were recognized in sections from the mid intestine from nine fish in each dietary group (three fish in each tank) using a monoclonal mouse antibody against proliferating cell nuclear antigen (PCNA; M0879, Dako, Glostrup, Denmark). Sections were routinely deparaffinized in xylene and rehydrated in graded alcohol baths before they were transferred to distilled water. Antigen retrieval was performed by autoclaving the slides in 0.01 M citrate buffer at 121°C for 15 min, and the slides were cooled to room temperature and transferred to phosphate-buffered saline (PBS) before inhibition of endogenous peroxidase with 0.05% phenyl hydrazine (P26252, Sigma-Aldrich, Milwaukee, Wisconsin, US) in PBS at 37°C for 40 min. The slides were then incubated in goat serum diluted 1:50 in 5% bovine serum albumin (BSA) in Tris-buffered saline (TBS) for 20 min to prevent nonspecific binding. Antisera against MHC class II, CD3ϵ and PCNA were diluted 1:600, 1:400 and 1:3000, respectively, in 1% BSA/TBS before incubation for 30 min. The secondary antibody and substrate chromogen were provided from the EnVision® System kit (K4009, Dako). The sections were counterstained with hematoxylin added acetic acid (Mayer’s hematoxylin) for 1 min and mounted with poly-vinyl alcohol mounting media (Ullevål Apotek, Oslo, Norway).

### Morphometric analysis

**Figure 4:**
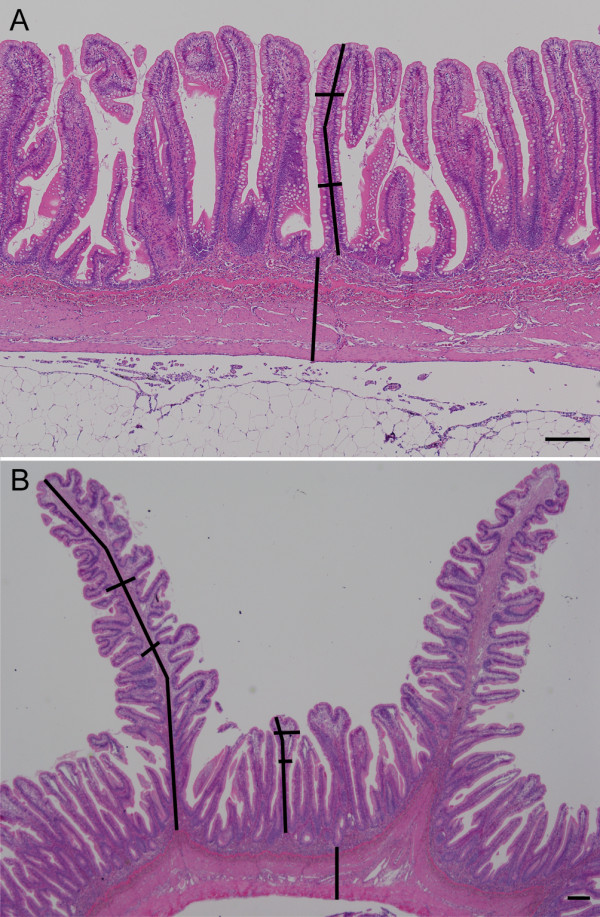
**Microphotographs showing measurements in sections of the mid (A) and distal (B) intestine.** The lines indicate how the height and width of the folds and the thickness of the wall were measured. Hematoxylin and eosin. Scale bar 200 μm.

Micrographs of intestinal sections from nine fish in each dietary group (three fish per tank) were captured and morphometric measurements were performed in the software NIS-Elements D version 3 (Nikon, Tokyo, Japan) using Nikon digital sight camera configured with a Nikon eclipse 80i microscope. The measurements were performed as previously described by Løkka *et al*. [28]. The height of the folds was measured from the fold apex to the bottom of the epithelium at the base of the folds, and both simple and complex folds were measured in the distal intestine. The width of the folds was assessed at two points in every fold, and the thickness of the intestinal wall was measured from beneath the epithelium at the base of the folds (simple folds in distal intestine) to the serosa (Figure 4). Five measurements of the fold height and wall thickness and ten measurements of the fold width in both intestinal segments were recorded for each individual.

### Gene transcription analysis

Tissues from the pyloric caeca, mid intestine and distal intestine (Figure 3) from nine fish in each dietary group (three fish per tank) were frozen in liquid nitrogen and stored at -80°C. The samples from the mid intestine were homogenized in Trizol using zirconium beads (4 mm) in a Retsch MM 310 homogenizer (Retsch GmbH, Haan, Germany). Subsequent addition of chloroform separated RNA from proteins and DNA, and RNA was then precipitated from the water phase by adding isopropanol. Furthermore, the RNA pellet was cleansed twice in ethanol and dissolved in RNase free water. A DNase treatment with DNA-free™ (Applied biosystems, Foster City, CA, USA) was performed on the RNA extract.

The samples from the pyloric caeca and the distal intestine were homogenized in Buffer RLT added mercaptoetanol using stainless steelbeads (5 mm) in a Retsch MM 300 homogenizer (Retsch GmbH). RNA was extracted with RNeasy Mini kit (QIAGEN, Hilden, Germany) using the protocol “RNeasyMini Animal Tissues and Cells Standard V3” followed by the protocol “Cleanup RNeasyMini RNA Standard V3” in a QIAcube (QIAGEN).

The concentration of RNA was measured using a Biospec-Nano (Shimadzu Corporation, Kyoto, Japan) or Nanodrop ND-1000 UV-Vis Spectrophotometer (NanoDrop Technologies, Wilmington, DE, USA). To verify acceptable quality of the RNA, 24 random samples were selected and tested on an Agilent 2100 Bioanalyzer (Agilent Technologies, Palo Alto, CA, USA). Total RNA was stored at -80°C.

The cDNA synthesis from 1 μg of total RNA was prepared with oligo(dT), random hexamer primers, M-MLV Reverse transcriptase (Promega, Madison, WI, USA) and RNase inhibitor (Promega) to prevent RNA degradation. Real-time PCR was carried out in 13 μl reactions using TaqMan Gene Expression Master Mix (Applied Biosystems, Carlsbad, CA, USA) with cDNA template corresponding to 15 ng of RNA in each reaction in a 7900HT fast real-time PCR system (Applied Biosystems) according to the producer’s instructions and running 40 cycles.

The following genes were analyzed by real-time RT-PCR: Cluster of differentiation 3ζ (CD3ζ), cyclooxygenase-2a (COX-2a), interleukin 1β (IL-1β), immunoglobulin M (IgM), immunoglobulin T (IgT), major histocompatibility class II (MHC class II), nucleotide-binding oligomerization domain-containing protein 2 (NOD2), transforming growth factor β (TGF-β), tumour necrosis factor α (TNF-α) and elongation factor 1A_B_ (EF1A_B_) as the reference gene [31] (Table 1). When possible, primers and probe were designed to span across intron sections. All analyses were performed in triplicates, and a control lacking the template for each master mix was always included in the experiments. The data were analysed using Sequence Detection Systems Software v2.3 (Applied Biosystems).

**Table 1:** Sequences of primers and probes used in real-time RT-PCR analysis

**Gene**	**Gene Sequence 5′ → 3′**	**GenBank accession no.**	**Pyloric caeca**	**Mid intestine**	**Distal intestine**
EF1A_B_	F-TGCCCCTCCAGGATGTCTAC	BG933853	18.19 ± 0.09	19.74 ± 0.28	18.90 ± 0.15
	R-CACGGCCCACAGGTACTG				
	P-FAM-AAATCGGCGGTATTGG-MGB				
CD3ζ	F-AACAGGGATCCAGAGAGTGCTG	BT060238	27.77 ± 0.16	27.74 ± 0.28	27.41 ± 0.20
	R-AAGGGACGTGTAAGTGTCGTCA				
	P-FAM-ACGGCACGCGATAATCGCAGGA-BHQ				
COX-2a	F-CAGATCGCTGGAAGGGTGG	AY848944	33.66 ± 0.31	33.32 ± 0.43	32.83 ± 0.35
	R-TCATGTTGAAGCGTTTCCTGTAG				
	P-FAM-AGCTAAGGCCCTGGAGCACAGC-BHQ				
IgM	F-TGTAAAGAGAGCAGACTGGGACAG	Y12456	25.60 ± 0.55	24.89 ± 0.60	24.63 ± 0.45
	R-GAGACGGGTGCTGCAGATATTC	Y12457			
	P-FAM-TGTTCCACGGCGCATTCAAAGATTT-BHQ				
IgT	F-CAGCAGTCTGCTGAAGGTC	GQ907004	29.91 ± 0.29	30.29 ± 0.50	28.00 ± 0.30
	R-GGTTCTGTTTTGGAGATCG	GQ907003			
	P-FAM-CTGCACCACACAGCTGTACTTGACC-BHQ				
IL-1β	F-GCTACCACAAAGTGCATTTG	AY617117	34.91 ± 0.42	33.91 ± 0.64	31.98 ± 0.33
	R-GAGGTTGGATCCCTTTATGC				
	P-FAM-CCATTGAGACTAAAGCCAGACCTGTAG-BHQ				
MHCII	F-CCACCTGGAGTACACACCCAG	X70165	20.95 ± 0.25	20.34 ± 0.35	20.49 ± 0.18
	R-TTCCTCTCAGCCTCAGGCAG				
	P-FAM-TCCTGCATGGTGGAGCACATCAGC-BHQ				
NOD2	F-GCATCCAGTGTGAGCACTTTCAG	EG915470	32.01 ± 0.19	31.55 ± 0.24	31.86 ± 0.23
	R-TTCATCTTCAGGAGGTGAGCG				
	P-FAM-CAAGCTAACTGATGCCTGCACAGAGTGC-BHQ				
TGF-β	F-TGGAGCTGAGTGAGGAGCAG	EU082211	34.04 ± 0.29	34.24 ± 0.41	33.46 ± 0.32
	R-ACCGCATCTCAGACATGTTG				
	P-FAM-TGTGGACCTCCTTTGCAAAGTATGC-BHQ				
TNF-α	F-GCAGCTTTATGTGCGGCAG	NM_001123589	36.38 ± 0.30	NA	35.47 ± 0.29
	R-TTTTGCACCAATGAGTATCTCCAG	NM_001123590			
	P-FAM-TGGAAGACTGGCAACGATGCAGGA-BHQ				

The mean Ct-values ± SEM for the fish oil group are given for each intestinal segment.

*NA*, Not applied.

### Calculations and statistical analysis

Databases for the results for morphometric measurements and real-time RT-PCR were established in Excel® for Windows, and statistical calculations and graphical presentation of real-time PCR results were performed using Prism 6.0 software (GraphPad Software, San Diego, CA, USA). Morphometric data within each dietary group were pooled before calculating the group mean.

Data are given as mean ± standard error of the mean (SEM). The data were analyzed for normality using a Shapiro Wilk’s test and for homogeneity of variance using a Brown-Forsythe’s test. For data that were non-normal and/or with non-homogeneous residuals, a log transformation was performed prior to one way analysis of variance (ANOVA) followed by Tukey’s multiple comparison test. The significance level was set to 0.05.

## Results

### Histology and immunohistochemistry

**Figure 5:**
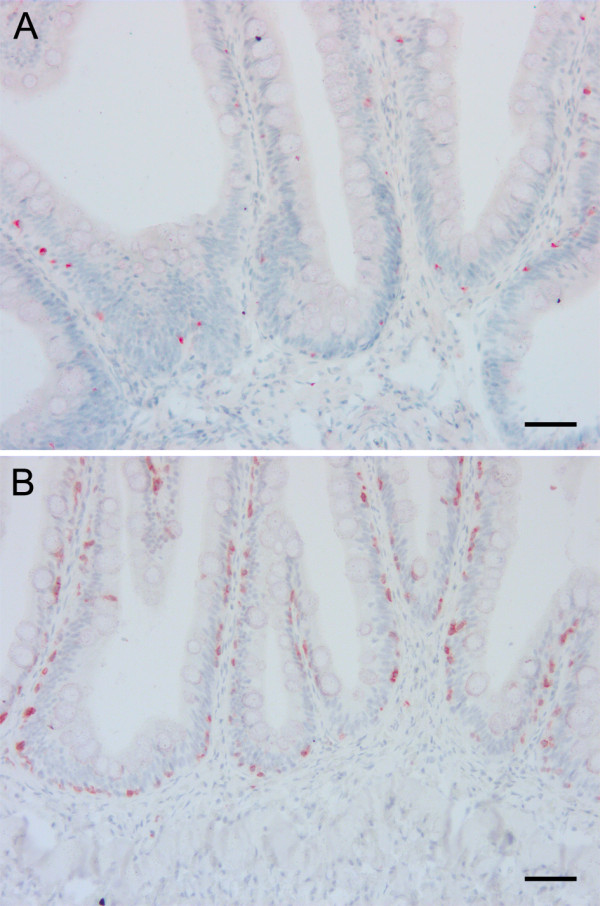
**Detection of antigen presenting cells and T lymphocytes in the mid intestine.** Microphotographs of mid intestinal sections from fish fed soybean oil stained with antisera against MHC class II **(A)** and CD3ϵ **(B)**. Positive cells visualized with red colour were found in the epithelium and only scattered cells were present in the lamina propria. The density and distribution of positive cells were independent of diet. Scale bar 50 μm.

Histological examination of the mid intestine did not show any obvious pathological changes in any fish, while there was shortening, widening and fusion of the simple folds with leucocyte infiltration in the lamina propria in the distal intestine in a few individuals without any obvious association with the different dietary groups. Examination of sections stained with antibodies against MHC class II and CD3ϵ showed positive cells both in the epithelium and in the lamina propria and with similar density and distribution of antigen presenting cells and T lymphocytes regardless of diet (Figure 5). The proliferation pattern of epithelial cells in the mid intestine as demonstrated by immunohistochemical staining with an antibody against PCNA did not differ between the dietary groups.

### Morphometric analysis

*Mid intestine*: The folds of the mid intestine were tallest in the fish oil group (1393 ± 36.4 μm), intermediate in the olive oil and rapeseed oil groups (1134 ± 31.2 μm and 1131 ± 42.4 μm respectively) and lowest in the soybean oil group (1012 ± 24.8 μm). The differences were highly significant (P < 0.0001) between the fish oil group and all vegetable oils groups. The folds of the mid intestine were widest in the soybean oil group (142.0 ± 5.4 μm) and most slender in the olive oil group (122.6 ± 4.3 μm) (P = 0.0249). There was no significant difference between the dietary groups regarding the wall thickness. The results from the morphometric analyses of the mid intestine are shown in Table 2.

**Table 2:** The height and width of folds and wall thickness in mid and distal intestine (μm)

**Mid intestine**				
	**FO**	**OO**	**RO**	**SO**
**Height of folds**	**1393 ± 36.4**^**a**^	**1134 ± 31.2**^**b**^	**1131 ± 42.4**^**b**^	**1012 ± 24.8**^**b**^
**Width of folds**	135.8 ± 4.8^ab^	**122.6 ± 4.3**^**a**^	129.2 ± 4.5^ab^	**142.0 ± 5.4**^**b**^
**Wall thickness**	732.2 ± 21.4^a^	652.3 ± 24.0^a^	652.5 ± 21.6^a^	694.0 ± 20.9^a^
**Distal intestine**				
	**FO**	**OO**	**RO**	**SO**
**Height of simple folds**	1240 ± 37.8^a^	1204 ± 39.7^a^	1221 ± 29.3^a^	1242 ± 35.7^a^
**Width of simple folds**	138.9 ± 5.3^a^	127.1 ± 5.5^a^	131.3 ± 5.9^a^	131.8 ± 5.3^a^
**Height of complex folds**	3456 ± 146.9^ab^	3280 ± 91.7^ab^	**3598 ± 100.2**^**a**^	**3123 ± 79.7**^**b**^
**Width of complex folds**	265.2 ± 10.8^a^	267.1 ± 10.7^a^	234.1 ± 7.8^a^	238.7 ± 10.1^a^
**Wall thickness**	**705.2 ± 20.4**^**a**^	**616.8 ± 22.8**^**b**^	643.3 ± 21.5^ab^	**621.4 ± 18.0**^**b**^

*FO*, fish oil, *OO*, olive oil, *RO*, rapeseed oil, *SO*, soybean oil.

^a,b^Significant differences (ANOVA, P < 0.05) between dietary groups in the same intestinal segment are denoted by different superscript letters. The values that differ significantly are highlighted in bold text.

*Distal intestine*: The complex folds were tallest in the rapeseed oil group (3598 ± 100.2 μm) and lowest in the soybean oil group (3123 ± 79.7 μm) (P = 0.0113), while the wall was thickest in the fish oil group (705.2 ± 20.4 μm) and thinnest in the olive oil group (616.8 ± 22.8 μm) (P = 0.0080). There was no significant difference between the dietary groups regarding the height of the simple folds or the width of either simple or complex folds. The results from the morphometric analyses of the distal intestine are shown in Table 2.

### Transcript levels of immune-related genes

Only small differences in the relative transcript levels of the various genes between the dietary groups were detected, and there was generally larger individual variation within a group than between the groups. Neither the transcript levels of the pro-inflammatory cytokines IL-1β and TNF-α, the intracellular receptor NOD2 nor the enzyme COX-2a that is involved in the synthesis of prostaglandins from fatty acids were significantly altered in any dietary group in any of the intestinal segments.

**Figure 6:**
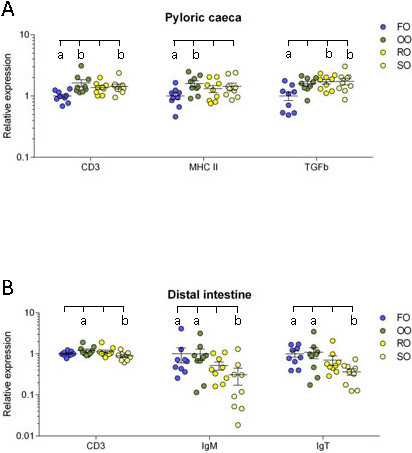
**Relative transcription levels of genes with significant differences between dietary groups.** The dot plots for CD3ζ, MHC class II and TGF-β in the pyloric caeca **(A)** and CD3ζ, IgM and IgT in the distal intestine **(B)** show the transcript levels for each individual with mean for each group as obtained by real-time RT-PCR analysis. Transcript levels are presented relative to the mean of the fish oil group. Error bars represent standard error of the mean. ^a,b^Significant differences (ANOVA, P < 0.05) between dietary groups are denoted by different letters above the dot plots. FO = fish oil, OO = olive oil, RO = rapeseed oil and SO = soybean oil.

*Pyloric caeca*: In the pyloric caeca, the olive oil group had significantly higher transcript levels of CD3ζ and MHC class II (P = 0.004 and 0.048) while the rapeseed oil group had a significantly higher transcript level of TGF-β (P = 0.022) and the soybean oil group had significantly higher transcript levels of CD3ζ and TGF-β (P = 0.033 and 0.017) compared to the fish oil group (Figure 6A).

*Mid intestine*: The transcript levels of the selected genes appeared to be more uniform in the mid intestine, 7and no significant differences between any dietary groups were detected in this intestinal segment.

*Distal intestine*: In the distal intestine, the transcript level of CD3ζ was significantly higher in the olive oil group than the soybean oil group (P = 0.035). The transcript levels of both IgM and IgT was significantly lower in the soybean oil group than in the fish oil group (P = 0.014 and 0.016) and the olive oil group (P = 0.015 and 0.038) (Figure 6B).

The underlying data for Figure 6A and B and the normalized transcript levels for all genes and dietary groups relative to the fish oil group are shown in Table 3. A disadvantage of calculating normalized transcription levels is that the differences between genes get lost, while the absolute Ct-values will reveal such differences. The Ct-values for MHC class II were relatively low (~18-20) in all intestinal segments of the fish in all dietary groups, indicative of a high transcript level of mRNA. The Ct-values for CD3ζ and the immunoglobulins were intermediate (~24-30), while the Ct-values for the selected cytokines, NOD2 and COX-2a were in general high (~31-37) and for some individuals beyond the detection limit. The mean Ct-values for all examined genes in the three intestinal segments for the fish oil group were included in Table 1.

**Table 3:** **Relative transcription levels of the immune-related genes normalized to EF1A**_**B**_

	**Pyloric caeca**				**Mid intestine**				**Distal intestine**			
	**FO**	**OO**	**RO**	**SO**	**FO**	**OO**	**RO**	**SO**	**FO**	**OO**	**RO**	**SO**
**CD3**	**1 ± 0.07**^**a**^	**1.63 ± 0.21**^**b**^	1.38 ± 0.10^ab^	**1.44 ± 0.13**^**b**^	1 ± 0.03^a^	1.22 ± 0.29^a^	0.99 ± 0.11^a^	0.96 ± 0.06^a^	1 ± 0.05^ab^	**1.21 ± 0.11**^**a**^	1.13 ± 0.11^ab^	**0.89 ± 0.07**^**b**^
**COX-2a**	1 ± 0.17^a^	1.56 ± 0.29^a^	1.36 ± 0.15^a^	1.28 ± 0.22^a^	1 ± 0.21^a^	0.98 ± 0.10^a^	1.08 ± 0.11^a^	1.20 ± 0.17^a^	1 ± 0.33^a^	0.85 ± 0.17^a^	1.19 ± 0.21^a^	0.85 ± 0.24^a^
**IgM**	1 ± 0.29^a^	1.37 ± 0.38^a^	0.80 ± 0.20^a^	0.97 ± 0.28^a^	1 ± 0.30^a^	0.93 ± 0.28^a^	0.72 ± 0.20^a^	0.95 ± 0.17^a^	**1 ± 0.40**^**a**^	**0.99 ± 0.30**^**a**^	0.52 ± 0.12^ab^	**0.31 ± 0.14**^**b**^
**IgT**	1 ± 0.17^a^	1.97 ± 0.61^a^	0.85 ± 0.12^a^	0.94 ± 0.17^a^	1 ± 0.22^a^	1.23 ± 0.18^a^	0.89 ± 0.22^a^	1.43 ± 0.30^a^	**1 ± 0.17**^**a**^	**1.09 ± 0.33**^**a**^	0.71 ± 0.18^ab^	**0.37 ± 0.07**^**b**^
**IL-1β**	1 ± 0.29^a^	0.94 ± 0.23^a^	0.63 ± 0.14^a^	0.91 ± 0.22^a^	1 ± 0.29^a^	1.51 ± 0.68^a^	0.90 ± 0.20^a^	0.89 ± 0.29^a^	1 ± 0.25^a^	1.26 ± 0.68^a^	1.07 ± 0.29^a^	0.92 ± 0.52^a^
**MHCII**	**1 ± 0.11**^**a**^	**1.59 ± 0.18**^**b**^	1.32 ± 0.17^ab^	1.44 ± 0.19^ab^	1 ± 0.11^a^	1.23 ± 0.26^a^	0.92 ± 0.15^a^	0.92 ± 0.14^a^	1 ± 0.14^a^	1.15 ± 0.13^a^	1.48 ± 0.18^a^	1.02 ± 0.15^a^
**NOD2**	1 ± 0.09^a^	1.31 ± 0.12^a^	1.32 ± 0.10^a^	1.21 ± 0.09^a^	1 ± 0.06^a^	0.98 ± 0.09^a^	0.92 ± 0.10^a^	0.87 ± 0.06^a^	1 ± 0.10^a^	1.23 ± 0.16^a^	1.90 ± 0.58^a^	0.96 ± 0.15^a^
**TGF-β**	**1 ± 0.15**^**a**^	1.59 ± 0.13^ab^	**1.71 ± 0.15**^**b**^	**1.74 ± 0.21**^**b**^	1 ± 0.16^a^	1.44 ± 0.27^a^	1.19 ± 0.21^a^	1.26 ± 0.11^a^	1 ± 0.16^a^	1.71 ± 0.41^a^	1.54 ± 0.27^a^	0.97 ± 0.33^a^
**TNF-α**	1 ± 0.14^a^	0.93 ± 0.19^a^	0.68 ± 0.11^a^	0.93 ± 0.16^a^	NA	NA	NA	NA	1 ± 0.13^a^	2.16 ± 1.31^a^	1.44 ± 0.28^a^	1.36 ± 0.75^a^

The data are presented as mean ± SEM relative to the mean of the fish oil group.

*FO*, fish oil, *OO*, olive oil, *RO*, rapeseed oil, *SO*, soybean oil, *NA*, Not applied.

^a,b^Significant differences (ANOVA, P < 0.05) between dietary groups in the same intestinal segment are denoted by different letters. The values that differ significantly are highlighted in bold text.

## Discussion

In this study, we have shown that fish fed diets where fish oil was largely replaced by three different vegetable oil blends had significantly shorter folds in the mid intestine compared with fish fed a diet with fish oil as the sole lipid source in a trial lasting for 28 weeks. The fold height decreased to a degree roughly corresponding to a decreasing n-3/n-6 fatty acid ratio of the feed, ie. the fish in the soybean oil group had the shortest folds. Additionally, the mid intestinal folds of the fish in the soybean oil group were significantly wider than of the fish in the olive oil group. In the distal intestine, the complex mucosal folds of the fish in the soybean oil group were significantly shorter than the folds of the fish in the rapeseed oil group, while the wall was significantly thicker for the fish in the fish oil group than the fish in the olive oil and soybean oil groups. Histological and immunohistochemical examination did not reveal any overt signs of inflammation in the lamina propria of the mid intestine. In the distal intestine, however, infiltration of inflammatory cells was observed in some individuals, but this could not be related to the dietary groups as fish in the fish oil group also were affected. Real time RT-PCR revealed only minor alterations in the transcript levels of the selected immune-related genes between dietary groups.

Dietary lipid sources have been reported to affect intestinal morphology in mammals. In weaning pigs, dietary supplementation with fish oil increased villus height in the small intestine combined with a decrease in transcript levels of inflammation related genes compared to a diet with corn oil [32]. Furthermore, dietary fatty acid composition has been reported to affect the height of intestinal villi in ileum in rats, the extent of the reductions increasing with increasing levels of n-6 fatty acids, ie. rats fed with fish oil had higher villi than those fed with olive oil and soybean oil [33]. Interestingly, rats fed with soybean oil had wider villi than the group given olive oil but not the group given fish oil, which is in agreement with our observations. The altered morphology was followed by a corresponding infiltration of mucosal lymphocytes [33].

In Atlantic salmon, shortening and widening of the simple mucosal folds of the distal intestine, in combination with infiltration of inflammatory cells in the lamina propria, has been repeatedly reported when feeding with soybean meal (SBM) and pea protein concentrate [23,24,34]. Starvation has also been described to mildly induce similar changes [23]. In humans, shortened intestinal villi and inflammation in the small intestine occurs in patients with coeliac disease caused by reaction to gluten proteins [35]. However, in the present study, histological investigation and immunohistochemical examination with antigen presenting cell and T lymphocyte markers did not show infiltration of inflammatory cells in the intestinal wall corresponding to the fold reduction pattern. Furthermore, there was no significant difference in the transcript levels of the investigated genes between either of the groups in the mid intestinal region. Combined, this indicates that the fold reductions observed in the mid intestinal region in the current study were not connected with a prolonged inflammatory response, but were probably caused by other factors.

In fish, the gastrointestinal microbiota is known to change with different feeding regimes [36,37] and more specifically with different lipid levels and different vegetable oils [38]. Alterations in intestinal microbiota are hence not to be neglected as a possible explanatory factor for the altered morphology observed.

Significant reductions of mucosal folds in the mid intestine of all vegetable dietary groups were observed, in contrast to the mildly affected distal intestine. This finding might be related to the fact that long chain fatty acids (LCFAs) mainly are absorbed in the pyloric caeca and mid intestine and only to a limited extent in the distal parts of the intestine [16,39]. Altering the composition of the LCFA in the feed can hence be speculated to cause most pronounced changes in the regions where these fatty acids are mainly absorbed.

Shortening of mucosal folds has been linked to altered proliferation pattern of the intestinal epithelium in Atlantic salmon; decrease in cell proliferation and apoptosis in smolt exposed to sublethal levels of inorganic mercury [40] and increase in cell proliferation in soybean meal induced enteropathy [36]. We did however not observe any differences in the proliferation pattern of epithelial cells in the mid intestine between any of the diet groups, even though lower turn-over in cell proliferation and apoptosis has been previously detected in Atlantic salmon fed with a diet where fish oil was completely replaced by plant oil [25].

Shortening of the mid intestinal folds probably reduces the total surface of the intestine and hence the absorption of nutrients, which may in turn influence the growth of the fish. A substantial proportion of starch and lipids is absorbed in the mid intestine [41]. Fish fed soybean oil were significantly shorter than the fish fed fish oil and somewhat (but not significantly) lighter than the other fish in the trial; results which were linked to reduced feed intake in the soybean oil group [22]. It cannot, however, be ruled out that the pronounced shortening of the folds in the mid intestine of fish fed soybean oil may be an additional factor contributing to the somewhat reduced growth in fish fed soybean oil. Furthermore, the difference in weight between the groups might have been more pronounced if not the fish fed fish oil as the sole lipid source had significantly reduced lipid digestibility due to high levels of dietary saturated fatty acids [22]. It cannot be ruled out that the reduced lipid digestibility in the fish oil group may have affected intestinal morphology as response to saturated fatty acids possibly being above an acceptable threshold level for Atlantic salmon. Overall, the shortened intestinal folds being most pronounced in the soybean oil group suggest that feeding with similar or higher levels of soybean oil as in the present trial might not be unproblematic for the production results.

In the pyloric caeca, the transcript levels of TGF-β and CD3ζ in the soybean oil group, the transcript level of TGF-β in the rapeseed oil group and the transcript levels of MHC class II and CD3ζ in the olive oil group was significantly higher than in the fish oil group. TGF-β is produced by cells of the innate immune system and by regulatory T lymphocytes and is both a pro- and anti-inflammatory cytokine that is involved in cell growth, migration, differentiation and apoptosis including inhibition of lymphocyte proliferation [42]. In SBM-induced inflammation in Atlantic salmon, transcription levels of TGF-β were reported up-regulated by 7-folds after 21 days, combined with a 20-folds up-regulation of IL-1β [43]. In the present trial, although significant, the differences observed in TGF-β transcript levels in the fish fed soybean oil and rapeseed oil compared to the fish fed fish oil was below two-fold, and there was no significant difference in IL-1β transcript levels between the groups; hence it is difficult to ascribe the differences in TGF-β transcript levels to an inflammatory process. The finding that neither the relative transcript levels of TNF-α, NOD2 nor COX-2a did vary significantly between the dietary groups in any of the intestinal segments also suggests that dietary lipids did not affect the degree of inflammation. The transcript levels of TNF-α and IL-1β in leucocytes from Atlantic salmon did not differ significantly between groups that were incubated in plasma with different n-3/n-6 ratio followed by stimulation with LPS, and a relative similar EPA/AA ratio in the cells was launched as an explanation to the lack of influence of fatty acid sources on inflammatory response [9]. A similar EPA/AA ratio in leucocytes may explain the relative stable transcript levels of cytokines regardless of diets in the current study too.

In contrast to mammals harboring mesenteric lymph nodes and distinct lymphoid follicles in the intestinal mucosa, the immune competent cells including antigen presenting cells as well as T and B lymphocytes are more diffusely spread in the intestinal tissue of teleosts like Atlantic salmon [44]. The moderately higher transcript levels of CD3ζ observed in the pyloric caeca of the olive and soybean oil group might indicate a slightly higher number of T lymphocytes as CD3ζ is part of the T cell receptor complex and expressed in all T lymphocytes [45]. MHC class II is in contrast expressed in antigen presenting cells that can present antigenic peptides to T lymphocytes and initiate the adaptive immunity [42]. In mammals, and presumably in teleosts, MHC class II is moreover expressed in intestinal enterocytes [46]. A higher transcript level of MHC class II might indicate a higher level of antigen presentation in the pyloric caeca of the olive oil group compared to the fish oil group. The differences in both CD3ζ and MHC class II transcript levels between the groups were however below two-fold and should be carefully interpreted. The higher transcript levels of certain genes in the plant oil groups compared to the fish oil group in the pyloric caeca could be linked to the high lipid absorption in this region [41]. However, this does not explain why we do not see a corresponding change in the transcript levels for these genes in the mid intestine as absorption rate for LCFAs are reported to be similarly high here [39].

The significantly lower transcript level of CD3ζ in the distal intestine of the soybean oil group compared to the olive oil group might in contrast to the pyloric caeca, indicate a lower number of T lymphocytes. Furthermore, the soybean oil group had significantly lower transcript levels of IgM and IgT than the fish fed both fish oil and olive oil. These immunoglobulins are expressed by different subpopulations of B lymphocytes in the teleost intestine [47] and are present both as membrane bound and secretory forms. Again, the differences were moderate, but summed together they suggest a difference in response to soybean oil between the pyloric cecea and distal intestine.

In mammals, replacement of n-3 fatty acids with n-6 polyunsaturated acids has been associated with intestinal inflammation and promotion of intestinal cancer [6,10,11]. Feed-induced intestinal carcinogenesis following inflammation has also been reported in brood stock Atlantic salmon [27], and it was speculated whether this partially could be related to replacement of fish oil with vegetable oils in the feed. Although the brood stock were exposed much longer to the commercial feed than the fish in the current 28 week-long trial, the results of the present study strongly suggest that partial replacement of fish oil with vegetable oils in the feed did not induce prolonged intestinal inflammation in Atlantic salmon.

The amount of fish oil still present in the feed might be of importance for the ability of the fish to cope with the increased amount of n-6 fatty acids. The regular feed composition used in the salmon industry has changed dramatically over the last decades as the lipid fraction has increased from 10% till about 35% [4]. This means that although vegetable oils constitute 80% of the lipid fraction as in the feeds of our study, the feed still contains approximately 1.4% EPA and DHA provided by the fish oil and fish meal included in the feed, which might be enough to sustain general intestinal health. It has been shown previously that 1% EPA and DHA in the feed is essential to attain good growth in fry [48]. A minimum proportion of EPA and DHA is considered to be required also for larger Atlantic salmon [49], however this has not yet been quantified.

## Conclusions

The folds in the mid intestine were significantly shorter in all groups of Atlantic salmon fed vegetable oils compared to the group fed fish oil. In the distal intestine, the complex folds were significantly shorter in the fish fed soybean oil compared to the fish fed rapeseed oil. Histological examination did not reveal clear difference in degree of inflammation related to dietary groups, and this finding was confirmed by real-time RT-PCR that only revealed moderate alterations in the mRNA transcript levels of selected immune-related genes.

The shortening of the intestinal folds was most pronounced in the fish fed soybean oil and might be associated with reduced intestinal surface and impaired nutrient absorption and growth. Kept together with significantly higher transcript levels of TGF-β and CD3ζ in the pyloric caeca and significantly lower transcript levels of IgM and IgT in the distal intestine in the fish fed soybean oil compared to the fish fed fish oil, it can be concluded that inclusion of high levels of soybean oil in the feed for Atlantic salmon should be done with caution.

## Competing interests

The authors declare that they have no competing interests.

## Authors’ contributions

TM did the sampling, planned the experiments, performed the histological examination, morphometric measurements, immunohistochemical stainings, parts of the cDNA synthesis and PCR and statistical analyses and drafted the manuscript, GL planned the experiments, performed immunohistochemical stainings, examination of immunohistochemical stainings, parts of the cDNA synthesis and PCR and statistical analyses and drafted the manuscript, JWN performed parts of the RNA isolation, cDNA synthesis and PCR, LA planned the experiments, designed the probes and supervised the PCR work, BT and GR designed and supervised the dietary experiment and samplings, and OBD, EOK and MK planned the experiments and supervised the study. Everyone commented on the manuscript and approved the final version.
